# Chloride channel-3 promotes tumor metastasis by regulating membrane ruffling and is associated with poor survival

**DOI:** 10.18632/oncotarget.2966

**Published:** 2014-12-11

**Authors:** Bin Xu, Xiaobao Jin, Ling Min, Qin Li, Lulu Deng, Hui Wu, Guixian Lin, Lixin Chen, Haifeng Zhang, Chunmei Li, Liwei Wang, Jiayong Zhu, Weizhang Wang, Fujiang Chu, Juan Shen, Hongzhi Li, Jianwen Mao

**Affiliations:** ^1^ Guangdong Key Laboratory for Bioactive Drugs Research, Guangdong Pharmaceutical University, Guangzhou, China; ^2^ School of Basic Medicine, Guangdong Pharmaceutical University, Guangzhou, China; ^3^ Department of Pharmacology and Department of Physiology, Medical College, Jinan University, Guangzhou, China; ^4^ School of Biosciences and Biopharmaceutics, Guangdong Pharmaceutical University, Guangzhou, China; ^5^ Cancer Center of Guangzhou Medical University, Guangzhou, China; ^6^ Department of Pharmacology, Guangdong Pharmaceutical University, Guangzhou, China

**Keywords:** chloride channel-3, tumor metastasis, membrane ruffling, cell migration

## Abstract

The chloride channel-3 (ClC-3) protein is known to be a component of Cl^−^ channels involved in cell volume regulation or acidification of intracellular vesicles. Here, we report that ClC-3 was highly expressed in the cytoplasm of metastatic carcinomatous cells and accelerated cell migration *in vitro* and tumor metastasis *in vivo*. High-grade expression of cytoplasmic ClC-3 predicted poor survival in cancer patients. We found that independent of its volume-activated Cl^−^ channel properties, ClC-3 was able to promote cell membrane ruffling, required for tumor metastasis. ClC-3 mediated membrane ruffling by regulating keratin 18 phosphorylation to control β1 Integrin recycling. Therefore, cytoplasmic ClC-3 plays an active and key role in tumor metastasis and may be a valuable prognostic biomarker and a therapeutic target to prevent tumor spread.

## INTRODUCTION

The initial step in tumor metastasis is the invasion of cancer cells into surrounding tissue and the vasculature. This requires chemotactic migration of cancer cells, steered by protrusive activity of the cell membrane [1]. Membrane ruffling of cells is a dynamic and rapid movement with irregular fluctuation of protrusion and withdrawal of the margin of the cell surface membrane [2]. A number of cytokines, including epidermal growth factor (EGF), have been shown to induce membrane ruffling [3]. Membrane ruffling has been shown to correlate with metastatic status and to be an indicator of cancer cell motility and metastatic potential [2, 4].

Membrane ruffles are often seen at the leading edge and on the dorsal surface of a migratory cell [5], and their structure, molecular composition, and the mechanisms leading to their formation remain largely unclear [6]. Actin remodeling is initially induced by cytokines to form membrane ruffles [7]. Signaling molecules that interact with the actin cytoskeleton, for example, small GTP-binding protein Rac, Ras, the adaptor protein Grb2, phosphatidyl inositol 3-kinase, phospholipases A_2_ and D_2_ and phorbol ester-responsive proteins may play important roles in ruffling [8, 9]. In recent years, several studies revealed that integrins (including β1 integrin) traffick to membrane ruffles or that circular dorsal ruffles play key roles in cell migration by regulating the formation of new focal adhesions [10-12]. However, the mechanism by which this occurs remains unknown.

ClC-3 is a member of the ClC voltage-gated Cl^−^ channel gene superfamily and reported to be localized in the plasma membrane and intracellular vesicles [13]. Membrane ClC-3 has been proposed as a key component of volume-activated Cl^−^ channels [14]. Vesicle ClC-3 may function as a Cl^−^ channel to facilitate endosomal acidification and loading of neurotransmitter [13, 15]. However, the role of ClC-3 as a constituent of native volume-activated Cl^−^ current (*I*_Cl,vol_) has become an issue of debate owing to inconsistent and conflicting data reported by some laboratories [16]. Some evidence supports the notion that ClC-3 may function as more than just a Cl^−^ channel [17, 18].

Studies have found that ClC-3 shows higher expression in cancer tissue such as glioma [19], lung [20], breast [21], and cervical tumors [22] compared to corresponding adjacent normal tissue. The migration and invasion of human glioma cells is regulated by ClC-3 [23]. The endocytosis of membrane ClC-3 channels inhibits glioma cell invasion *in vitro* and *in vivo* [24]. Previously, we reported that ClC-3 may play roles in the migration of nasopharyngeal carcinoma CNE-2Z cells and HeLa cells [25, 26]. Either the invasion or migration of cancer cells is a key early event in the formation of metastases. These results imply that ClC-3 may have an important role in tumor metastasis.

ClC-3 is thought to act as a volume-activated Cl^−^ channel to regulate cell shape changes during cell migration [27, 28]. However, ClC-3 is predominantly expressed in the cytoplasm and nuclei of tumor cells such as glioma D54–MG cells [29], CNE-2Z cells[30] and HeLa cells[31]. Therefore, we need to determine whether cytoplasmic and nuclear ClC-3 modulates cell migration via other mechanisms, besides acting as a volume-activated Cl^−^ channel.

In this study, we investigated the non-ion channel mechanisms by which ClC-3 mediates membrane ruffling and cell migration and promotes tumor metastasis.

## RESULTS

### Cytoplasmic ClC-3 Overexpression Correlated Positively with Human Tumor Metastasis

**Figure 1:**
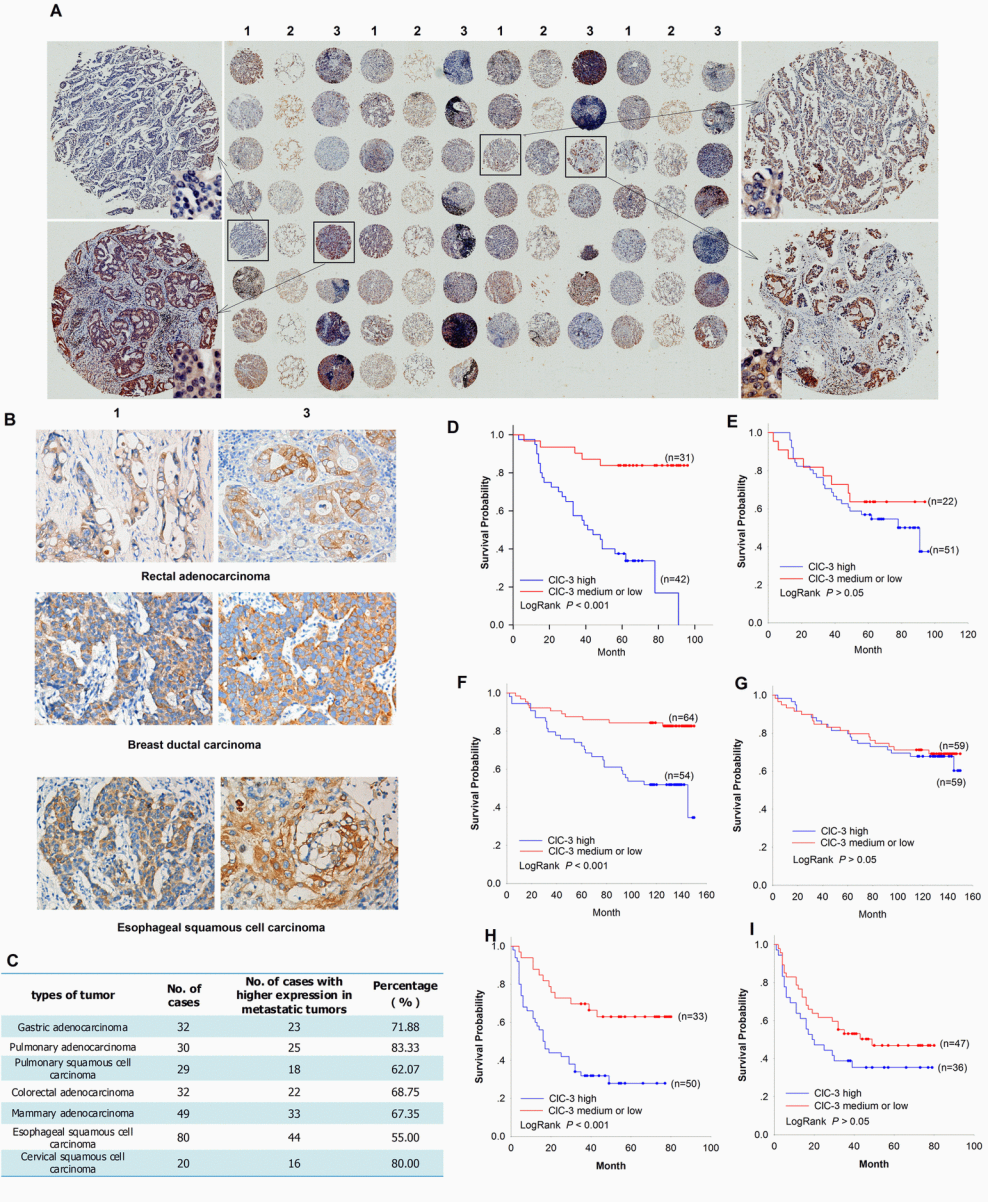
Association between ClC-3 Expression and Tumor Metastasis or Survival in Cancer Patients (A-C) Analyses of ClC-3 Expression Difference between Primary and Metastatic Tumors. Overview of immunohistochemical staining of ClC-3 in a tissue microarray section containing 30 pairs of primary pulmonary adenocarcinoma and their matched lymph node metastatic tumors (A) .1: primary tumor; 2: adjacent non-neoplastic tissue; 3: matched lymph node metastatic tumor. Representative immunohistochemical images for ClC-3 sampled from tissue microarray of rectal adenocarcinoma, breast ductal carcinoma and esophageal squamous cell carcinoma (B). Summary of higher expression percentage of cytoplasmic ClC-3 in metastatic tumors compared to the corresponding primary tumors (C). (D-I) Association between cytoplasmic or nuclear ClC-3 expression and survival in primary carcinomas. Kaplan–Meier survival estimates for high- and intermediate- or low-grade cases of lung (D), breast (F) and liver (H) cancer regarding cytoplasmic ClC-3 expression. Kaplan–Meier survival curves were generated to assess differences between high- and intermediate- or low-grade nuclear ClC-3 expression cases of lung (E), breast (G) and liver (I) cancer.

Our previous studies found that down-regulation of ClC-3 expression reduce cancer cell migration [26, 32]. These suggested that elevated expression of ClC-3 may be associated with an increased metastatic capacity of primary human cancer. To test this hypothesis, we evaluated ClC-3 expression in several types of cancers including lung, stomach, colon, rectum, esophagus, breast and cervix carcinoma by immunostaining. In 272 pairs of primary tumors and their matched metastatic tumors, ClC-3 expression could be detected mainly in the cytoplasm and some in both cytoplasm and nucleus (Figure 1A, B and S3A). Comparing the expression between primary tumors and their matched metastatic tumors, cytoplasm expression of ClC-3 in 181 of 272 (69.8%) pairs of tumors was clearly higher in metastatic tumors than in their corresponding primary tumors (Figure 1A-C).

### Cytoplasmic ClC-3 is a Prognostic Biomarker for Survival in Tumor Patients

Because metastatic potential generally affects the long-term survival of patients after curative resection of the primary tumor, we analyzed the effect of ClC-3 expression on cancer-related survival in a cohort of 274 tumor patients (including 73 lung adenocarcinoma, 118 breast adenocarcinoma and 83 liver cancer) with a median follow-up of 6 months (range 0.8–13.4 months). One patient was lost to follow-up. Indeed, the log rank test demonstrated that tumors with the high cytoplasmic ClC-3 expression (IRS score ≥9) were associated with short overall patient survival, whereas patients with tumors displaying intermediate- or low- grade cytoplasmic ClC-3 expression (IRS score < 9) showed a better clinical outcome (Figure 1D,F,H). However, we did not find that a change in the expression of nuclear ClC-3 was associated with patients' survival in any of the three types of tumors (Figure 1E, G, I). Taken together, cytoplasmic ClC-3 expression seems to be a valuable prognostic biomarker for cancer patients.

### Involvement of ClC-3 in Mouse Tumor Metastasis Models

**Figure 2:**
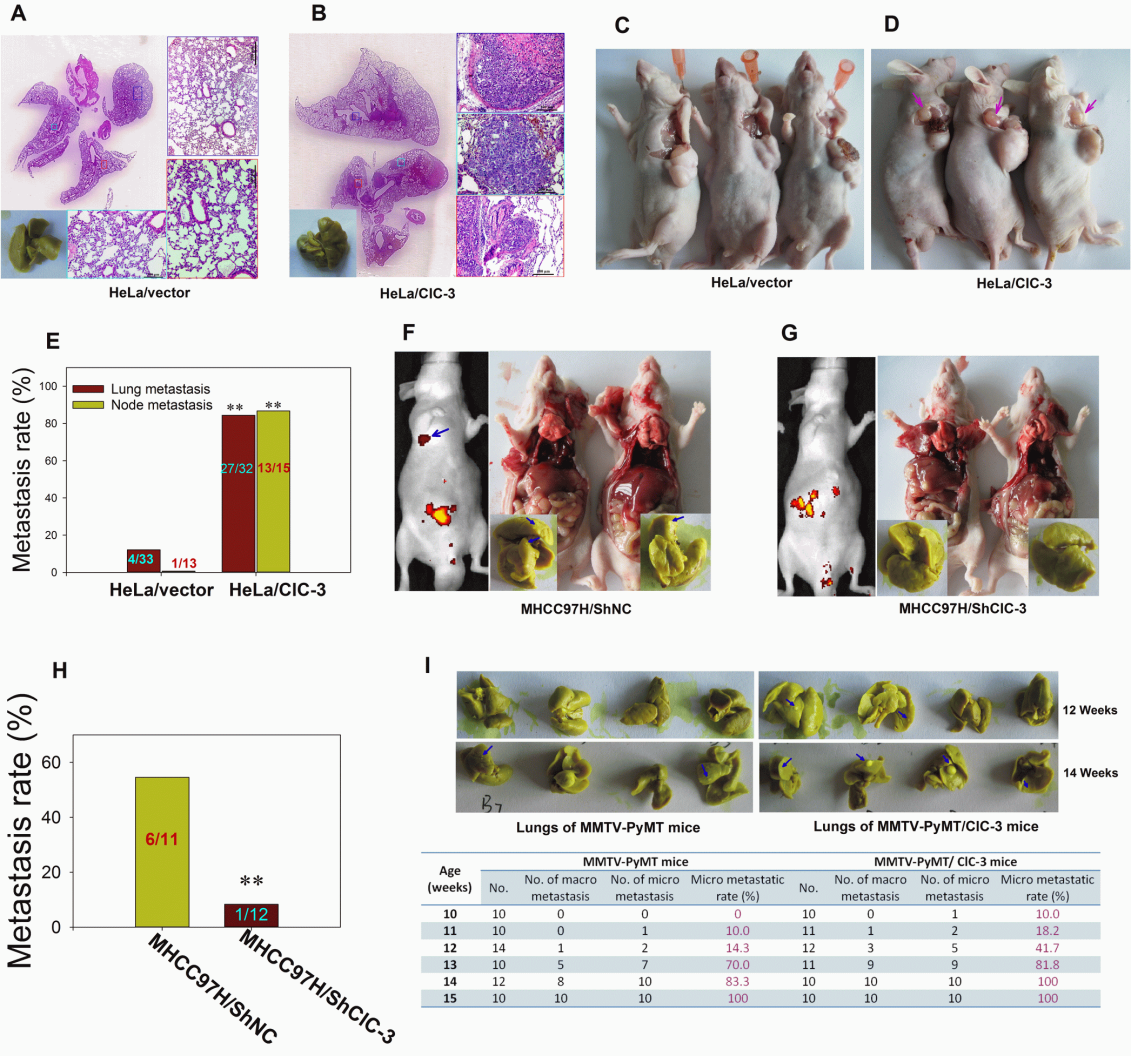
Overexpression or Knockdown of ClC-3 Promotes or Degrades Metastasis in Animal Models of Experimental Pulmonary and Spontaneous Metastases (A and B) Lung metastasis formation at 8 weeks after the injection of HeLa/PcDNA3.1 (A) or HeLa/ClC-3 cells (B) into the tail vein of nude mice. (C and D) Macroscopic aspects of axillary lymph node metastases at about 1-cm xenograft formation after the subcutaneous injection of HeLa/PcDNA3.1 (C) or HeLa/ClC-3 (D) cells into nude mice. (E) Histograms depicting the microscopic or macroscopic metastastic rate in each group are shown. Data are mean ± SEM. ** *P* < 0.01 *vs* corresponding control groups. (F-H) Bioluminescence (left) and autopsy (right) imaging after liver orthotopic implantation of high-metastatic potential MHCC97H cells infected with the negative control (GFP alone, shNC, F) or siRNA lentiviruses (ClC-3-siRNA1-GFP, shClC-3, G). Macroscopic metastastic rate in each group are presented in (H). Data are mean ± SEM. (I) Overexpression of ClC-3 in MMTV-PyMT mice (spontaneous mammary tumor model) by crossing with ClC-3 transgenic mice accelerates lung metastasis. Representative lungs from MMTV-PyMT/ClC-3 transgenic mice were isolated at 12 or 14 weeks and stained with Bouin's solution (upper). The number of lungs with metastases was determined using dissecting microscopy and histological examination of H&E-stained sections. The percentage of mice with metastases developed in lung at different phase is shown.

**Figure 3:**
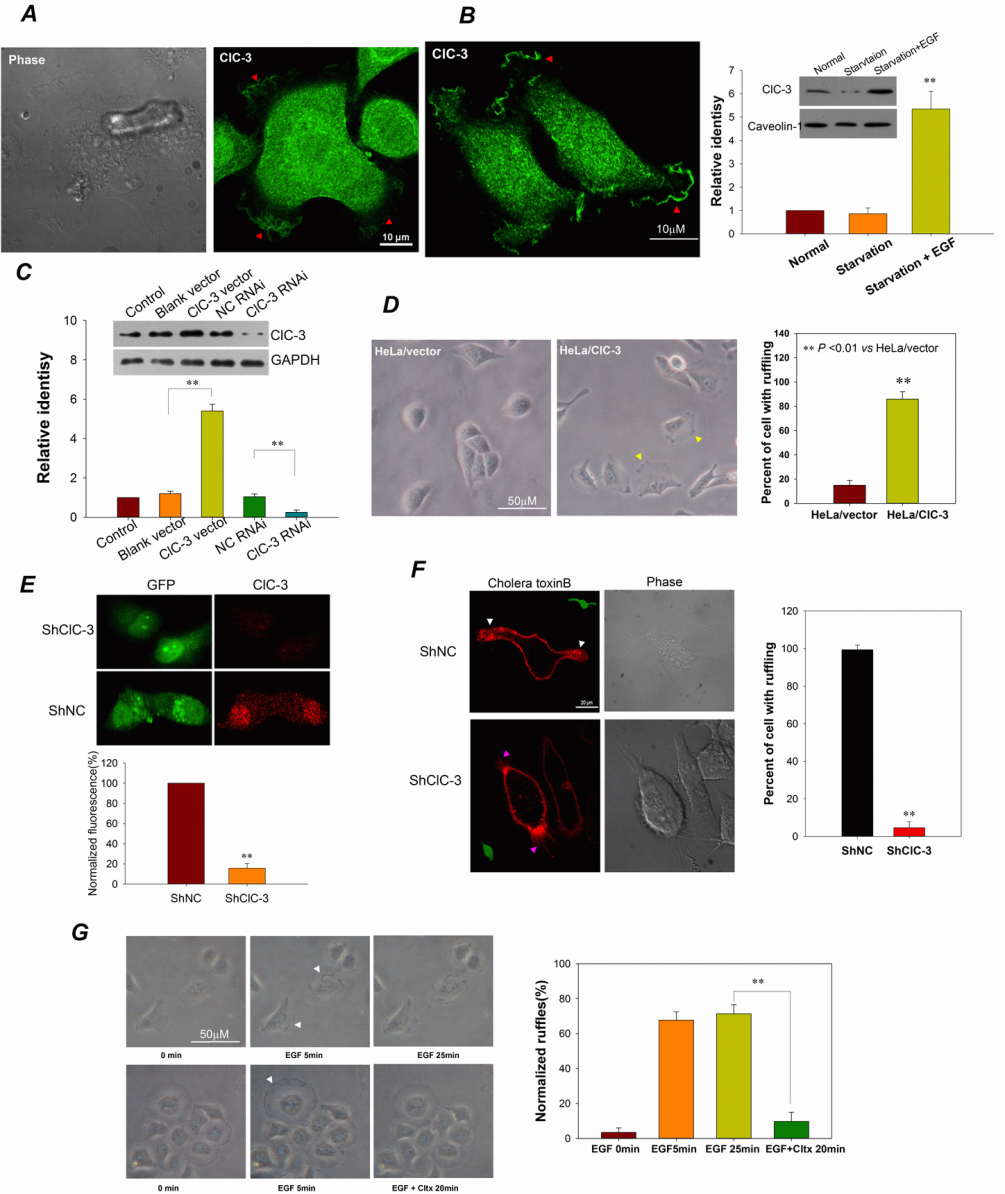
ClC-3 Is Involved In Membrane Ruffling (A and B) ClC-3 gathers at membrane ruffles in the leading edge of lamellipodia of migrating HeLa cells without (A) or with (B, left) EGF stimulation. Cells were fixed with paraformaldehyde and incubated with anti-ClC-3 antibody and Alexa Fluor 488-conjugated secondary antibody. The red arrows indicate membrane ruffles. Western blot analysis (B, right) shows that the EGF stimulation significantly increased the amount of ClC-3 on the membrane surface. See also Movie S1. (C and D) Up-regulation of ClC-3 expression promotes membrane ruffling in HeLa cells. (C) Western blots analysis to demonstrate ClC-3 overexpression by stably transfecting with ClC-3/pcDNA 3.1 plasmid and ClC-3 knockdown by transiently transfecting with ClC-3 RNAi. (D) Bright-field photographs of live cells at 20 min after subculturing of cells. **P<0.01 *VS* HeLa/vector; n=3 for Western blots analysis and 4 with >100 cells for bright-field ruffling observation. Data are mean ± SEM. (E and F) Down-regulation of ClC-3 expression prevents membrane ruffling in HeLa cells. (E) immunofluorescence analysis to demonstrate ClC-3 knock-down by transfection of shClC-3 vectors (pGPU6/GFP-ClC-3 shRNA). (F) Membrane ruffling was visualized by staining with Alexa Fluor-555 cholera toxin B (CTXB) in live cells. Ruffling was lost in cells transfected with shClC-3 (green) when EGF stimulation.**P<0.01 *VS* negative control shRNA (shNC); n=3 with >15 cells. Data are mean ± SEM. White arrows indicate membrane ruffles. Pink arrows mark filopodia. (G) Chlorotoxin inhibits EGF-induced membrane ruffling. (Left) Bright-field photographs of live cells to observe membrane ruffling. White arrows indicate membrane ruffles. (Right) Membrane ruffling (normalized to cell perimeter) was quantified. **P<0.01; n=3 with >20 cells.

We asked whether ClC-3 function is required during metastasis in a mouse model. There was a low incidence of metastasis with few lung tumor nodules in mice inoculated intravenously with HeLa cells (Figure 2A). However, overexpression of ClC-3 in the HeLa cell line increased lung tumor burden as compared with the HeLa vector cells (Figure 2A, B and 3C). Similarly, up-regulation of ClC-3 expression markedly increased the incidence of lymph node metastasis compared with control stable HeLa cells in the xenograft mouse model (Figure 2C-E). We next investigated the effect of ClC-3 expression knockdown on the lung metastasis potential of high metastatic potential MHCC97H cells. The results demonstrated that there was about 54.5% lung metastasis incidence when MHCC97H cells were embedded in situ into liver. Down-regulation of ClC-3 expression significantly decreased the incidence of metastasis and number of lung tumor nodules (Figure 2F-H and S3C).

To further confirm the *in vivo* promoting activity of ClC-3 on tumor metastasis, we generated ClC-3 transgenic mice and crossed them with MMTV-PyMT spontaneous mammary tumor model mice. The resulting double transgenic mice (MMTV-PyMT/ClC-3) developed breast tumors with simultaneous expression of ClC-3 throughout the body. Compared with MMTV-PyMT mice, mammary cancer in MMTV-PyMT/ClC-3 mice exhibited earlier metastatic tendency and higher lung metastatic rate (Figures S1 and Figure 2I).

### ClC-3 Is Necessary for Membrane Ruffle Formation

ClC-3 was found to be involved in cell migration [26, 32]. To dissect the functions of ClC-3 in caner metastasis, we first examined its subcellular location in migrating HeLa cells. We found that ClC-3 gathered at membrane ruffles in the leading edge of lamellipodia of migrating cells (Figure 3A, B; Movie S1). EGF stimulation induced membrane ruffling and increased the mount of membranous ClC-3 (Figure 3B). To further test whether ClC-3 is involved in the formation of membrane ruffles, we determined the effect of changes in ClC-3 expression on membrane ruffling. The results demonstrated that HeLa cells stably overexpressing ClC-3 showed significantly more membrane ruffling (Figure 3C, D). Moreover, silencing ClC-3 expression by transfection with ClC-3-specific shRNA almost completely abolished EGF-induced membrane ruffling (Figure 3E, F). Chlorotoxin has been shown to induce nearly complete endocytosis of membrane ClC-3 channels [24]. Light microscopy showed that chlorotoxin almost completely eliminated EGF-induced membrane ruffles, indicating that endocytosis of ClC-3 of membrane ruffles inhibited membrane ruffling (Figure 3G). Collectively, these data suggest that ClC-3 is necessary for membrane ruffle formation.

### Regulation of Membrane Ruffle Formation by ClC-3 Is Independent of its Volume-activated Cl^−^ Channel Properties

ClC-3 is suggested to be a component of the volume-activated Cl^−^ channel in the plasma membrane. To further test whether ClC-3 is involved in membrane ruffling through its volume-activated chloride channel properties we next examined the effects of chloride channel blockers NPPB and tamoxifen on membrane ruffle formation. Our data showed that after the volume-sensitive Cl^−^ current was activated by 47% hypotonic stimulation (160 mOsmol/L), both blockers almost completely (and similarly) inhibited the currents. However, neither NPPB nor tamoxifen prevented or abrogated membrane ruffling induced by EGF (Figure S2A-E). This hints that ClC-3 of membrane ruffles did not work as volume-activated Cl^−^ channels. In addition to the cell membrane, ClC-3 is also located in intracellular vesicle membranes, functioning as Cl^−^ channels to facilitate endosomal acidification. We wondered whether vesicle membrane ClC-3 is involved in membrane ruffling as a Cl^−^ channel. We next observed the effect of intracellular dialysis of the non-specific Cl^−^ channel blocker NPPB or tamoxifen on membrane ruffling. After 20 min of intracellular dialysis by adding 200 μM NPPB or 40 μM tamoxifen into the pipette solution, EGF-induced membrane ruffles still occurred (Figure S2F). Several residues (serine 51, serine 362 and tyrosine 284) play roles in ClC-3 channel function [33, 34]. The phosphorylation of serine residues (serine 51 and serine 362) induced by PKC inhibited volume activated chloride currents. The tyrosine 284 phosphorylation in the rat ClC-3 channel (Tyrosine 342 in the human ClC-3 channel) was found to be an important molecular mechanism for ClC-3 channel activation. A non-phosphorylatable mutation (Y284F) abolished the ClC-3-mediated increment of Cl^−^ current and Cl^−^ efflux induced by hypotonic solution [34]. In order to observe the effect of the decrease of volume activated chloride currents on membrane ruffling under the conditions of unchanged ClC-3 expression, we then observed that the effects of Y342F mutation of ClC-3 on Cl^−^ current mediated by ClC-3 and membrane ruffling. The results show that Y342F mutation obviously inhibited the Cl^−^ current (Figure S2G) induced by hypotonic solution but not stopped the formation of membrane ruffling induced by EGF (Figure S2H). Together, these data demonstrate that the roles of ClC-3 in membrane ruffling are independent of its volume-activated Cl^−^ channel properties.

### ClC-3-mediated Membrane Ruffling Is Related to Cancer Cell Migration

**Figure 4:**
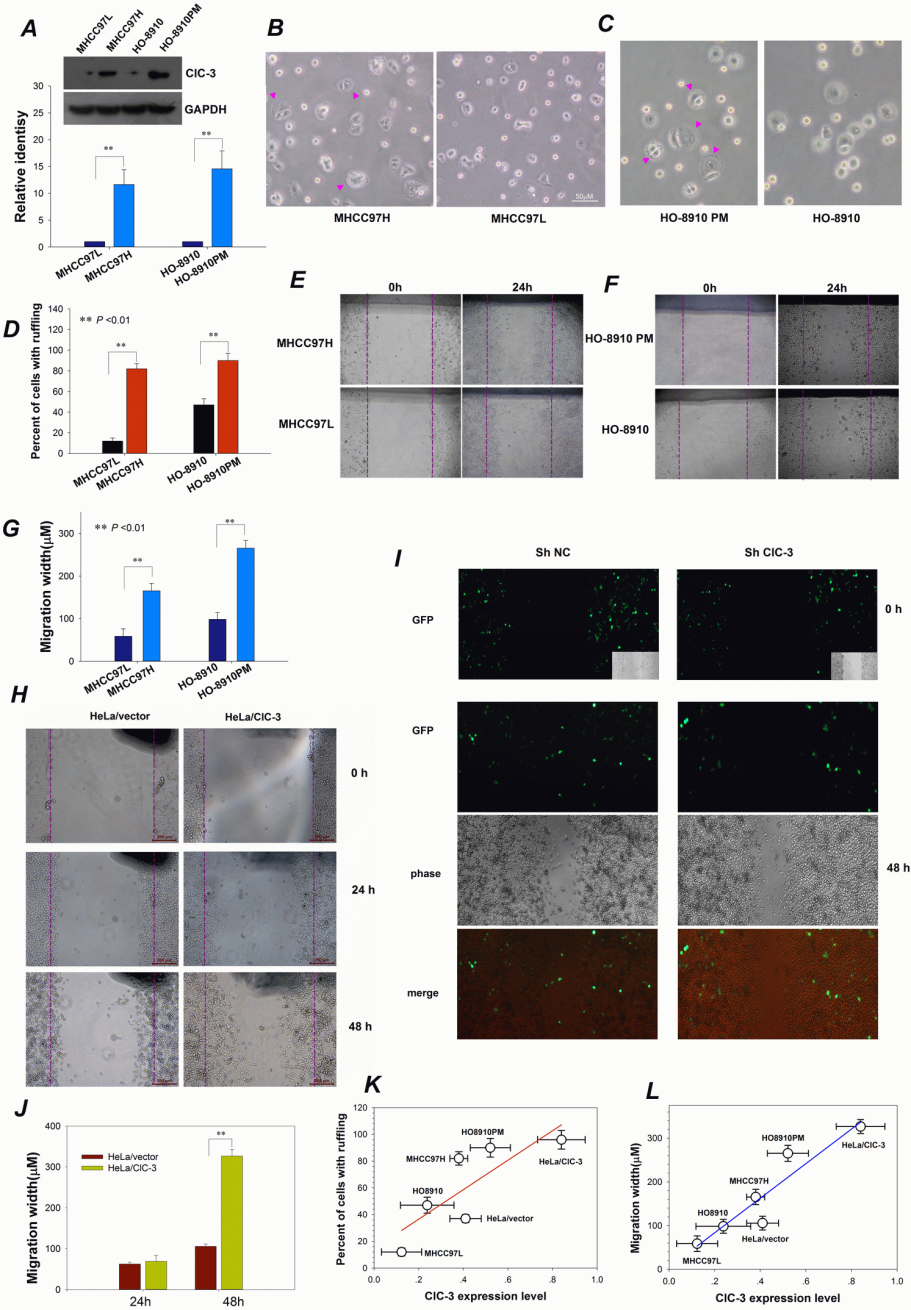
ClC-3-mediated Membrane Ruffling Is Related to Cancer Cell Migration (A) Representative Western blot probed for total ClC-3 to detect expression differences between high- (MHCC97H and HO8910PM) and low- (MHCC97Land HO8910) metastatic potential cancer cell lines.**P<0.01; n=3. Data are mean ± SEM. (B-D) Comparison of membrane ruffling capability between high- and low-metastatic potential liver (B) and ovarian (C) cancer cell lines and quantification of multiple visual fields (n = 10, D). Bright-field photographs of live cells were taken at 20 min after subculturing of cells. Pink arrows indicate membrane ruffles. (E -G) Observation of cell migration ability for high- and low-metastatic potential liver (E) and ovarian (F) cancer cell lines. Confluent monolayers were scratched and then cultured in the medium along with EGF (10 ng/ml) for different time. Data shown in (G) are mean ± SEM. (H-J) Altering ClC-3 expression affects cell migration in an *in vitro* wound assay. Representative photographs of scratch wound-healing motility assays obtained from HeLa cells (HeLa/ClC-3) stably transfected with PCDNA3.1-ClC-3 vectors (H) or HeLa/ClC-3 cells transiently treated with shRNA against ClC-3 (shClC-3, I). Average migratory width of three independent experiments is shown (J, data are mean ± SEM, ** *P* < 0.01). (K) Positive correlation between ClC-3 expression and membrane ruffling. Percentage of cells with ruffling is plotted against the level of ClC-3 protein expression. Fitting the data with the equation, f = y0 + ax, results in a linear correlation coefficient (r) of 0.98 (*P* < 0.01; y0 = 4.5, a = 395.3). (L) A positive correlation between ClC-3 expression and cell migration is obtained by plotting the migration rate against the ClC-3 expression level under the same treatments and by fitting the data with the equation f = y0 + ax. Fitting yields a linear correlation coefficient of r = 0.81 (*P* < 0.05; y0 = 14.5, a = 110.2).

As shown above, ClC-3 expression promotes membrane ruffle formation. Membrane ruffling has been shown to be an indicator of tumor cell motility and metastatic potential [2]. Accordingly, cells with high metastatic potential should have much more membrane ruffles and higher migration potential. To validate this, differences in ClC-3 expression, membrane ruffling ability and cell migration potential between high- and low-metastatic potential cell lines were assessed. Both high-metastatic MHCC97H and HO-8910PM cells possessed higher ClC-3 expression, more membrane ruffles and greater migration distance than corresponding low-metastatic MHCC97L and HO-8910 (Figure 4A-G). The effects of up-regulation or down-regulation of ClC-3 expression on cell migration were also evaluated. HeLa cells with stably overexpressing ClC-3 had faster movement. However, silencing ClC-3 expression stopped the HeLa/ClC-3 cells' locomotion (Figure 4H-J). Analysis of the relationship to each other of ClC-3 expression and membrane ruffling and cell migration showed that the former were positively correlated with the latter two (Figure 4K, L). The positive relationship provides additional evidence for the involvement of ClC-3-mediated membrane ruffling in cancer cell migration. Together, these data indicate that ClC-3-regulated membrane ruffling is closely related to cancer cell migration.

### ClC-3 Contributes to the Recycling of β1 Integrin

**Figure 5:**
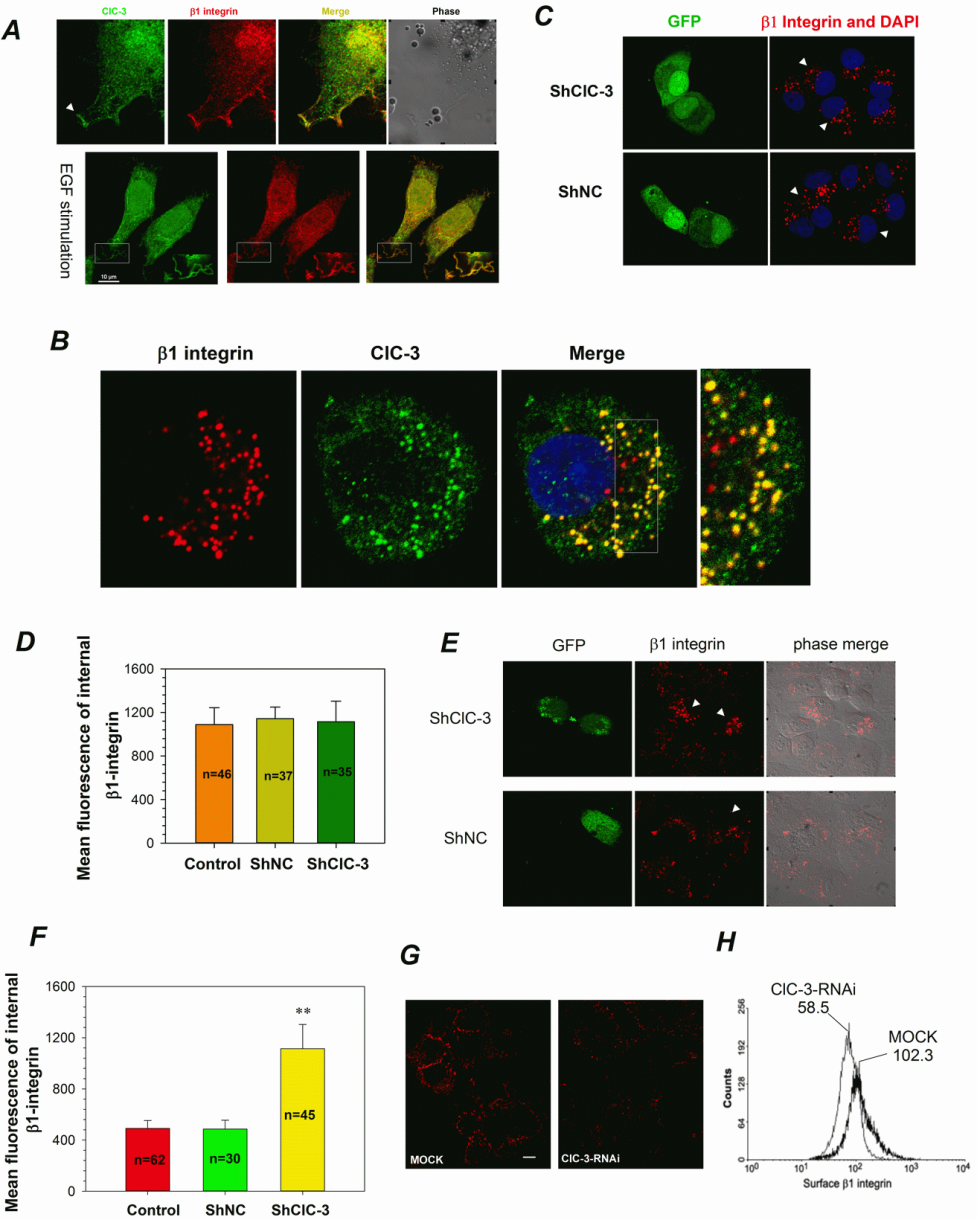
ClC-3 is Involved in β1 Integrin Recycling (A) Immunofluorescence of HeLa cells stained for ClC-3 (green) and β1 Integrin (red) indicates that ClC-3 colocalized with endogenous β1 integrin in ruffles (arrow or rectangle) in HeLa cells with or without EGF stimulation. The Pearson coefficient is 0.79±0.08 for without EGF stimulation and 0.71±0.04 for EGF stimulation (mean ± SEM, n=3 with 8 and 12 cells). (B) Indirect immunofluorescence indicates that ClC-3 and internalized β1-integrin colocalized together perfectly in the cytoplasm. The Pearson coefficient is 0.73±0.05 (mean ± SEM, n=3 with 10 cells). Surface β1 integrin was labeled with anti-β1 integrin antibody and the cells then incubated at 37°C for 2 h (pulse) to induce integrin-antibody complexes to be internalized. (C and D) Representative images(C) and quantitative analysis (D) show that down-regulation of ClC-3 expression did not affect membrane β1 integrin internalization. Before internalization assay HeLa cells were treated with shClC-3 or shNC for 48h. # *P* >0.05 *VS* shNC. Data are mean ± SEM. White arrows point transfected cells (green). (E-I) ClC-3 knockdown affects β1 integrin recycling in HeLa cells. Following β1 integrin internalization, recycling of anti-β1-integrin/β1-integrin complexes to the plasma membrane occurred by stimulation with serum. HeLa cells on plates (G-I) or coverslips (E and F) were mock-treated or treated with shClC-3 or ClC-3-RNAi. Immunofluorescence for detecting the fixed cells (E, arrows mark transfected cells) and mean fluorescence analysis of internal β1-integrin (F) suggests that internalized β1-integrin accumulates in the perinuclear region in ClC-3-knockdown cells transfected with shClC-3. β1-Integrin recycling to plasma membrane in live HeLa cells was visualized by immunofluorescence (H) and quantitatively measured by a flow cytometry recycling assay (I). ClC-3 knockdown (G) clearly impairs β1 integrin recycling to the plasma membrane. **P<0.01 *VS* shNC or mock. Data in (F and G) are mean ± SEM.

β1 Integrin has been revealed to be involved in membrane ruffling by internalizing, recycling and clustering in membrane ruffles [10]. We wondered whether ClC-3 has roles in β1 integrin trafficking during membrane ruffle formation. Using double labeling with immunofluorescence detection, we first found that ClC-3 colocalized with endogenous β1 integrin in ruffles in HeLa cells with or without EGF stimulation (Figure 5A). To follow β1 integrin trafficking, we next used 12G10 anti-β1 integrin antibody to label surface β1 integrin [35] and then incubated the cells at 37°C for 2 h (pulse) to induce integrin-antibody complexes to be internalized. Indirect immunofluorescence showed that ClC-3 and internalized β1-integrin colocalized closely in the cytoplasm (Figure 5B). These results imply that ClC-3 may mediate membrane ruffling by regulating β1 integrin internalizing and recycling. To confirm this, the effects of down-regulation of ClC-3 expression by transfecting with ClC-3 shRNA or SiRNA on β1 Integrin internalizing and recycling were examined. The results showed that internalized β1 integrin in ClC-3 shRNA-transfected cells mainly accumulated in the perinuclear region, and there was no difference with mock-treated cells (Figure 5C, D). This hints that ClC-3 may not be involved in the internalization of β1 integrin. Following 1 h incubation with medium containing 20% fetal bovine serum (FBS) to stimulate internalized β1 integrin recycling, most of the internalized β1 integrin in mock-treated cells were recycled to the membrane. However, cells treated with ClC-3 shRNA or SiRNA displayed sustained perinuclear β1 integrin aggregation and little immunostaining of recycled membrane β1 integrin (Figure 5E-H).

In a variety of cultured cancer cells, such as CNE-2Z [30], HeLa [31] and D54–MG [29], ClC-3 has been found to localize in the nucleus. Using IHC in several types of cancer tissue including lung cancer and breast cancer and normal tissues such as stomach and esophagus epithelium, we found that ClC-3 also expressed in the nucleus (Figure S3A). The non-existence of nuclear membranous organelles suggests that ClC-3 may work as a non-ion channel protein. Accordingly, we presumed that besides membranous organelles, ClC-3 may also localize in cytoplasmic non-membranous organelles and/or interstitial cytoplasm, which is involved in the recycling of β1 integrin. Using a plasma membrane (including cell membrane and endomembrane) and nuclear protein isolation kits and Western blot assay, we found that in the majority of cells, total ClC-3 was non-membranous ClC-3 in the cytoplasm (Figure S3B). Moreover, stable down-regulation of ClC-3 expression by about sevenfold with transfection of ClC-3 shRNA viral vector was mostly reduced cytoplasmic non-membranous ClC-3. Transfection clearly impaired the recycling of β1 integrin but did not affect the membranous ClC-3 and volume-activated Cl^−^ current (Figure S3C-E). Collectively, these data show that non-membranous cytoplasmic ClC-3 may play a crucial role in β1 integrin recycling.

### ClC-3 Mediated Recycling of β1 Integrin by Inducing Keratin 18 Phosphorylation

**Figure 6:**
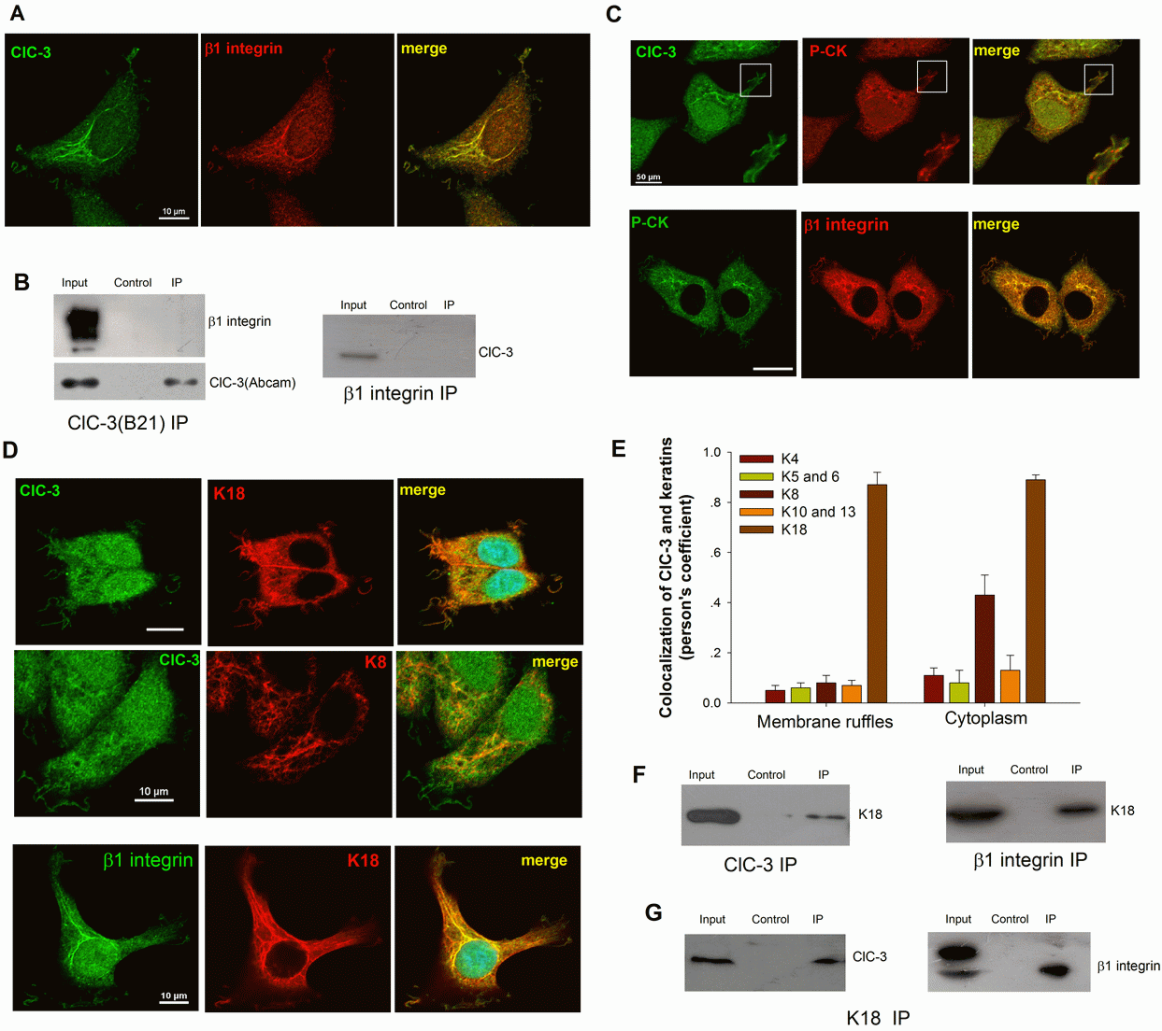
ClC-3 Regulates β1 Integrin Recycling through Binding to K18 (A) Besides membrane ruffles, ClC-3 and β1 integrin also colocalize in cord-like structures in the cytoplasm of HeLa cells stimulated with EGF. The Pearson coefficient is 0.87±0.04 (mean ± SEM, n=3 with 15 cells). (B) ClC-3 and β1 integrin do not bind to each other. Left: Immunoblots of co-IP with anti-ClC-3 antibody (B21) from extract of HeLa cells treated with EGF, probed with antibodies against β1 integrin or ClC-3 (Abcam). Right: co-IP with anti-β1 integrin antibody from the same extract probed with anti-ClC-3. (C) Immunofluorescence staining for ClC-3, β1 Integrin and pan-cytokeratin (P-CK) indicates that ClC-3 (Pearson coefficient: 0.87±0.05 for ruffles and 0.89±0.02 for cytoplasm, n=3 with 16 cells) or β1 integrin (Pearson coefficient: 0.89±0.02 for ruffles and 0.91±0.03 for cytoplasm, n=3 with 14 cells) colocalizes with P-CK in membrane ruffles and cytoplasm in HeLa cells stimulated with EGF. Bar: 20μM. (D and E) ClC-3 or β1 Integrin (Pearson coefficient: 0.78±0.04 for ruffles and 0.92±0.06 for cytoplasm, n=3 with 13 cells) colocalize with K18 in membrane ruffles and cytoplasm in cells with EGF stimulation. (D) Representative Immunofluorescence images. (E) A Pearson's coefficient was calculated to estimate the degree of co-localization of the different keratins with ClC-3. The results represent >20 cells from n = 2 independent experiments. (F) Co-IP experiment with anti-β1 integrin (right) or ClC-3 (left) antibody from the extract of ruffling HeLa cells (EGF treatment) probed with K18 showed that β1 Integrin or ClC-3 is binding to K18 respectively. (G) Co-IP with anti-K18 antibody from the extract of ruffling HeLa cells (EGF treatment) probed with anti-ClC-3 or β1 Integrin.

We next sought to understand how ClC-3 regulates β1 integrin recycling. We first tested whether ClC-3 and β1 Integrin colocalized and combined in the cytoplasm. With EGF stimulation, ClC-3 and β1 integrin were found to colocalize in cord-like structures in the cytoplasm (Figure 6A). However, ClC-3 and β1 integrin were not bound to each other (Figure 6B). This suggests that ClC-3 does not directly regulate β1 integrin recycling. What is then the mediator between ClC-3 and β1 integrin? The cytoskeleton, including microtubules, microfilaments and intermediate filaments, is known to play key roles in intracellular protein traffic. We then speculated that one or more of cytoskeleton components may be involved in the regulation of β1 integrin recycling by ClC-3. Immunofluorescence detection showed that except for keratin, ClC-3 did not colocalize with microtubules, microfilaments or vimentin in the cytoplasm during membrane ruffling (Figure 6C and S4A-C, F). Keratin 18 (K18) was identified as the most possible keratin molecule involved in the regulation of β1 integrin recycling by immunofluorescence colocalization observation in HeLa cells and different types of cancer tissues (Figure 6D, E, Figure S4D-F). Co-IP analysis confirmed and showed that K18 and ClC-3 were associated with β1 integrin, respectively (Figure 6F, G). Together, these data suggest that ClC-3 may mediate β1 integrin recycling by regulating K18 organization.

**Figure 7:**
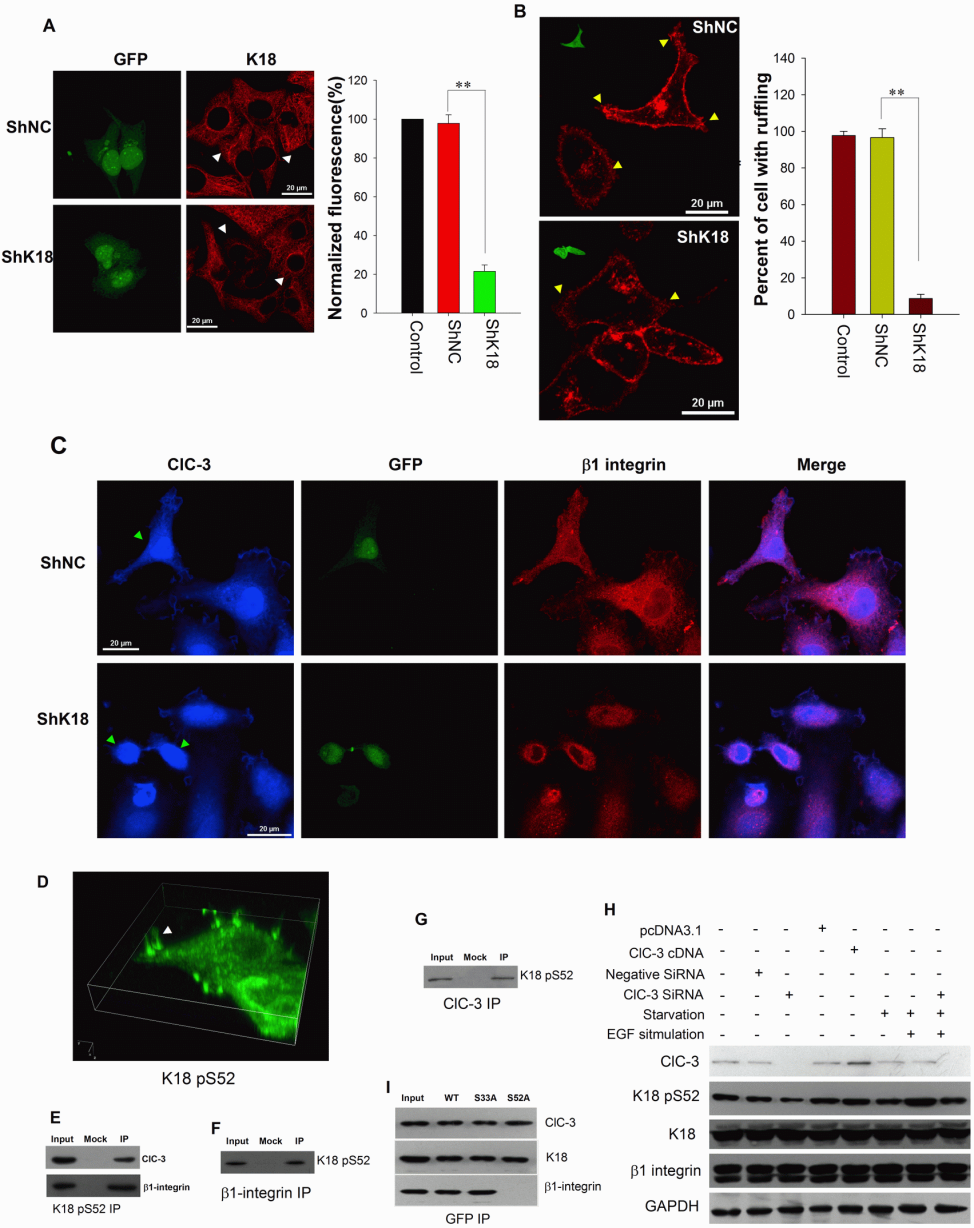
ClC-3 Mediates Recycling of β1 Integrin by Inducing Keratin 18 Phosphorylation (A) Immunofluorescence images (Left) and evaluation of fluorescence intensity of K18 (right) in HeLa cells treated with shK18 (pGPU6/GFP-K18 shRNA) or shRNA negative control (shNC). **P<0.01; n=3 with >15 cells. Data are mean ± SEM. White arrows mark transfected cells. (B) K18 knockdown by transfection of shK18 inhibits membrane ruffling induced by EGF. (Left) Fluorescent CTXB labeling photographs of live cells. *Insets* in images show transfected cells by detection of GFP. Yellow arrows indicate membrane ruffles. (Right) Percentage of cells with membrane ruffling was calculated in control or transfected conditions (data are mean ± SEM, **P < 0.001 n=3 with >50 cells). (C) K18 down-regulation prevents K18 and ClC-3 trafficking between cytoplasm and membrane leads them to gather in perinuclear region. After HeLa cells transfected with shK18 or shNC were stimulated by EGF for 5 min immunofluorescence analysis for ClC-3 and β1 integrin carried out. Green arrows point transfected cells. (D) 3D reconstruction of subcellular localization of Ser-52 phosphorylated K18 (K18 pS52) in ruffling cells. Immunofluorescence of Ser-52 phosphorylated K18 in EGF-stimulated HeLa cells was detected with an anti- K18 pS52 antibody and an Alexa Fluor 488-conjugated secondary antibody. White arrows indicate membrane ruffles. See also Movie S2. (E) Co-IP experiment with anti-K18 pS52 antibody from extract of ruffling cells probed with anti-ClC-3 and β1 Integrin. (F and G) Co-IP with anti-ClC-3 (F) or anti-β1 Integrin (G) antibody from extract of ruffling cells probed with anti-K18 pS52. (H) Manipulation of ClC-3 expression by overexpression or knockdown of ClC-3 results in altered Ser-52 phosphorylation of K18. (I) Effects of blocked phosphorylation of K18 at Ser-52 on interaction between ClC-3 or β1 integrin and K18 in EGF-stimulated HeLa cells by mutation of Ser-52 to Ala. Cells were transfected with vectors expressing wild-type (WT) or Ser-33 and Ser-52 (Ala) mutant GFP-K18.

To further confirm the roles of K18, we examined the effects of down-regulation of K18 expression on membrane ruffling and β1 integrin and ClC-3 trafficking. The results showed that down-regulation of K18 expression with transfection of K18 shRNA almost completely inhibited EGF-induced membrane ruffling and prevented β1 integrin and ClC-3 trafficking between the cytoplasm and membrane, leading them to gather in the perinuclear region (Figure 7A-C).

K18 reorganization is dependent on Ser52 phosphorylation [36]. ClC-3 may mediate β1 integrin recycling via K18 reorganization by promoting its phosphorylation. Using a specific antibody for phosphorylated Ser52 K18 (K18 pS52), we then investigated the relationship between ClC-3, β1 integrin and phosphorylated keratin 18. We found that K18 pS52 also gathered at membrane ruffles (Figure 7D; Movie S2). Furthermore, the three proteins colocalized in the cytoplasm in different types of cancer tissues, and K18 pS52 associated with ClC-3 and β1 integrin, respectively (Figure S5 and Figures 7E-G). Up-regulation of ClC-3 expression accelerated the phosphorylation of K18 Ser52, and down-regulation halted phosphorylation. Furthermore, the phosphorylation of Ser52 was highly elevated during membrane ruffling with EGF stimulation, but transfection with ClC-3 SiRNA significantly impaired the positive effect of EGF (Figure 7H). Finally, we tested whether K18 Ser52 phosphorylation plays a role in K18 binding to ClC-3 or β1 integrin proteins. As shown in Fig. 7I, In the presence of EGF, binding of K18 to β1 integrin was abolished if a K18 Ser52→Ala mutant was transfected, but binding of K18 to ClC-3 was not altered. Binding of K18 to either β1 integrin or ClC-3 did not change if a K18 Ser33→Ala mutant was transfected. Together, these data suggest that ClC-3 may mediate β1 integrin recycling via K18 reorganization by promoting Ser52 phosphorylation.

## DISCUSSION

Although several signaling molecules, including Rac, Ras, Arf and Grb2, have been reported to regulate the formation of ruffling [8, 9], proposed mechanisms for manipulating ruffling and the effect of these factors on cell migration and tumor metastasis are only beginning to emerge. In this study, we observed that ClC-3, a member of the ClC chloride channel gene family, accumulated at membrane ruffles in the leading edge of lamellipodia of migrating cells and on the dorsal surface of cells treated with EGF. Over- or down-expression of ClC-3 clearly promotes or prevents the formation of membrane ruffles. Moreover, endocytosis of membrane ruffles ClC-3 induced by chlorotoxin eliminated EGF-induced membrane ruffles. These results show that ClC-3 is necessary to promote membrane ruffle formation.

Membrane ruffling is closely related to cancer cell motility and metastatic potential [2]. This indicates that ClC-3 may have roles in cancer cell migration and metastasis. To verify this notion we analyzed the relationship between ClC-3 expression, membrane ruffling ability and cell migration potential between high- and low-metastatic potential cell lines. We also observed the effects of altering ClC-3 expression on cancer cell migration ***in vitro*** and tumor metastasis *in vivo*. Our results showed that ClC-3 expression was positively correlated with membrane ruffling ability and cell migration potential, respectively, and that over- or down-expression of ClC-3 promoted or inhibited cancer lymph node and distant metastasis *in vivo*. Further experiments with crossing established ClC-3 transgenic mice with MMTV-PyMT spontaneous mammary tumor model mice, and detecting the difference in ClC-3 expression between human primary and their matched metastatic tumors confirmed that up-regulation of ClC-3 expression plays a crucial role in cancer cells obtaining high metastatic potential. These data strongly support the notion that ClC-3 has a critical role in tumor metastasis by mediating membrane ruffling.

What is the role of ClC-3 in mediating membrane ruffling? ClC-3 localizes in the plasma membrane and intracellular vesicles. Since Duan and coworkers declared in 1997 that ClC-3 may be the long-sought volume-activated Cl^−^ channel [37], most researchers focused on the physiological and pathological function of ClC-3 as a volume-activated Cl^−^ channel. Furthermore, ClC-3 was found to gather on invadopodia and may function as volume-activated Cl^−^ channels to facilitate invasive cell shrinkage [38]. Accordingly, we first hypothesized that ClC-3 mediates membrane ruffling by regulating volume-activated Cl^−^ channels to change the cell shape of migratory cells. Inconsistent findings, however, were obtained in this study. Neither NPPB nor tamoxifen prevented or abrogated membrane ruffling induced by EGF, although both chloride channel blockers almost completely inhibited the volume-activated Cl^−^ currents. Consistent with our findings, several research groups have questioned the function of ClC-3 as a volume-activated Cl^−^ channel, owing to the lack of effect of altered ClC-3 expression on volume-activated Cl^−^ current in cell lines [18, 39, 40] and cells isolated from three independent ClC-3 knockout (*Clcn3*^−/−^) mice [41-43]. These findings suggest that ClC-3 may not be involved in membrane ruffling as a volume-activated Cl^−^ channel.

ClC-3 also localizes in the intracellular vesicle membrane and works as a Cl^−^ channel to facilitate vesicle acidification. We next considered that vesicle membrane ClC-3 may function as Cl^−^ channels to regulate membrane ruffling. But the non-effects of intracellular dialysis of non-specific Cl^−^ channel blocker NPPB on membrane ruffling didn't support this notion. However, due to the lack of effective means of detecting volume activated chloride currents of intracellular vesicle, the present results can not completely rule out vesicle ClC-3 participates and function as a Cl^−^ channel in the regulation of membrane ruffling.

It is now recognized that cell surface ClC-5, a close ClC-3 homolog, functions as a key component, independent of its role in ion transport, in the assembly of the macromolecular complex involved in protein endocytosis [44, 45]. This gives us a hint that ClC-3 may have a similar role and play as a critical regulatory molecule in the formation of membrane ruffles. In the experiment of EGF-induced membrane ruffling, we found that ClC-3 showed a cord-like distribution in the cytoplasm (Figure 6A). We speculated that ClC-3 may be involved in the membrane-cytoplasm trafficking of important molecules related to membrane ruffling. β1 integrin internalization and recycling have been revealed to play a key role in membrane ruffling [10, 11]. We next found that ClC-3 and β1 integrin colocalized in ruffles and in cytoplasm with EGF stimulation or with β1 integrin internalization. After excluding the involvement of ClC-3 and β1 integrin in the regulation of endocytosis, we found that silencing the expression of ClC-3 significantly inhibited β1 integrin recycling. These data suggest that ClC-3 regulates membrane ruffling by modulating β1 integrin recycling.

**Figure 8:**
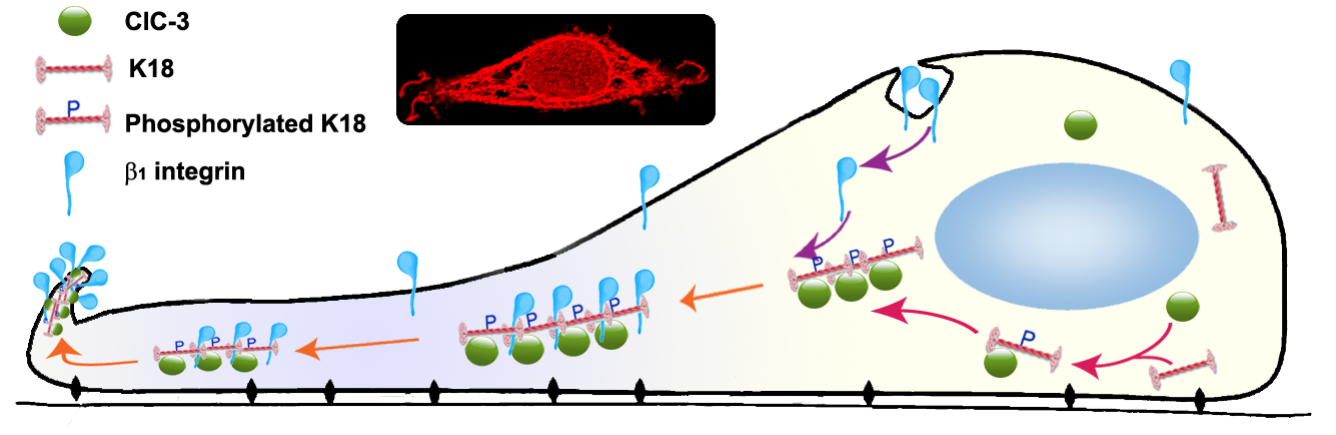
Proposed Model for Regulation of ClC-3 on Recycling of β1 Integrin ClC-3-mediated β1 integrin recycling via K18 reorganization by promoting Ser-52 phosphorylation. Under EGF stimulation, cytoplasmic ClC-3 first binds to K18 and initiates its reorganization to pave roads by promoting Ser52 phosphorylation, and then internalized β1 integrin transport to membrane ruffles along the paved way by binding to phosphorylated K18. *Insets* representative confocal photomicrograph of ClC-3 immunofluorescence in a ruffling HeLa cell induced by EGF stimulation.

How does ClC-3 regulate β1 integrin recycling? Upon phosphorylation by Akt, ACAP1 directly binds to β1 integrin on endosomal membranes to promote integrin recycling [46]. ClC-3 may directly bind to β1 integrin to regulate its trafficking. But the Co-IP results showed that ClC-3 did not directly interact with β1 integrin. β1 integrin trafficking in the cytoplasm occurs along two traditional routes (a Rab4-mediated or a Rab11-regulated route) [47] or non-conventional pathway [48-50]. We found that ClC-3 did not colocalize with Rab4 or Rab11 in the cytoplasm after internalization of β1 integrin. This indicated that ClC-3 may regulate recycling of β1 integrin via a non-traditional route. We next focused on the roles of cytoskeleton on β1 integrin recycling mediated by ClC-3. After excluding several cytoskeletal proteins (tubulin, actin and vimentin), we finally determined that keratin 18 (K18) functioned as a bridge in ClC-3-mediated regulation of β1 integrin recycling. Researchers have found that PKC-mediated phosphorylation of cytoskeleton vimentin is a key process in integrin trafficking through the cell [49]. Consistent with this, our further results demonstrated that knockdown of ClC-3 abated the increase in K18 Ser52 phosphorylation induced by EGF and that K18 Ser52→Ala mutant prevented the binding of ClC-3 to K18. These findings indicated that ClC-3-dependent Ser52 phosphorylation of cytoskeleton K18 was necessary for β1 integrin trafficking to membrane ruffles. Ser52 phosphorylation can trigger K18 reorganization [36]. Together, our data paint a picture for the mechanism of ClC-3-mediated β1 integrin recycling. In this picture, under EGF stimulation, cytoplasmic ClC-3 first binds to K18 and initiates its reorganization to pave roads by promoting Ser52 phosphorylation, and then internalizes β1 integrin for transport to membrane ruffles along the paved way by binding to phosphorylated K18 (Figure 8).

Over the past decade, researchers have found that ClC-3 is highly expressed in human cervical cancer [22], lung cancer [20], breast cancer [21] and malignant glioma [19]. Multiple research groups including ours have reported that ClC-3 is involved in the cell cycle [29], apoptosis [51], proliferation [25], migration [32] and invasion [23] of tumor cells. Our present study also suggests that ClC-3 plays a key role in tumor metastasis. Together, these results suggest that the expression of ClC-3 may be highly related to the clinical prognosis of some caners. The log rank test for censored survival data showed that cancers with high-grade cytoplasmic ClC-3 expression were associated with short overall patient survival, whereas patients with cancers displaying intermediate- or low-grade cytoplasmic ClC-3 expression showed a better clinical outcome. Changes in expression of nuclear ClC-3 were not associated with cancer patients' survival. Thus, cytoplasmic ClC-3 expression seems to be a valuable prognostic biomarker for cancer patients. The function of highly expressed ClC-3 in the cytoplasm as a signal molecule to promote tumor metastasis via membrane ruffling by controlling K18-dependent recycling of β1 integrin possibly contributes to the main theoretical support for prognostic evaluation.

Many questions remain unclear for the function of ClC-3 in tumor metastasis. For example, what causes metastatic cancer cells to express ClC-3 at high levels? What's the mechanism by which the binding of ClC-3 to K18 results in elevated phosphorylation of K18? Does binding activate protein kinase C (PKC) epsilon to phosphorylate K18 [52]? How does ClC-3 enhance β1 integrin binding to phosphorylated K18? What is the function of the ClC-3/K18/β1 integrin complex gathered at membrane ruffle? Is aggregation a preparation for macropinocytosis and recycling again of β1 integrin via traditional ways to repopulate newly formed focal adhesions on the ventral surface of lamellipodia in migratory cancer cells [12]? Answering these questions will lead to a deeper and more comprehensive understanding of the mechanisms of membrane ruffling, cell migration and tumor metastasis.

In conclusion, we report that ClC-3 is highly expressed in the cytoplasm of metastatic cancer cells and accelerates cell migration and tumor metastasis by modulating membrane ruffling. We revealed that ClC-3 mediates membrane ruffling independently of its volume-activated Cl^−^ channel function by directly regulating K18 phosphorylation to control β1 integrin recycling. We believe that cytoplasmic ClC-3 could be a valuable prognostic biomarker for cancer patients and a therapeutic target to restrict tumor spread.

## METHODS

### Cell Culture, Establishment of Stable Cell Lines

HeLa cells, MHCC97H and MHCC97L cells were cultured as previously described [31, 53]. HO8910 and HO8910PM cells were purchased from the Cell Bank of Type Culture Collection of the Chinese Academy of Sciences (Shanghai, China) and cultured as described elsewhere [54]. The cell lines were characterized by DNA fingerprinting analysis using short tandem repeat markers. HeLa cells with stable over-expression of ClC-3 (HeLa/ClC-3 cell) was obtained by transfecting with ClC-3/pcDNA 3.1 plasmid (kindly provided by Dr. Debrah Nelson, University of Chicago, Chicago, IL) and using geneticin (G418) as previously described [55]. Stable knock-down of ClC-3 in HeLa cells was achieved by expression of shRNA from lentivirus vector pLLU2G-shCLCN3 under the control of the CMV promoter for stable expression (Cyagen Bioscience Inc., Guangzhou, China)

### Immunofluorescence and Western Blot

Experiments were performed according to protocols previously described [31]. Samples were incubated overnight at 4^0^C with different dilutions for immunofluorescence with primary antibodies against ClC-3 (1:50, Abcam), β1 integrin (1:100, BD Pharmingen), α-tubulin (1:50, Abcam), vimentin (1:100, Boster), K18 (1:100, Boster) and K18 pS52 (1:50, Abcam). All antibodies were diluted to 1:1000 for immunoblotting.

### Electrophysiological Experiments and Intracellular Dialysis

Membrane Cl^−^ currents were recorded with the patch clamp technique in the whole-cell voltage-clamp mode with a patch clamp amplifier (L/M-EPC-7, List Electronic, Darmstadt, Germany) according to the method previously described [56]. Patch pipettes had resistances of 4-6 MΩ when filled with the standard intracellular solution (70 mM N-methyl-D-glucamine chloride (NMDG-Cl), 1.2 mM MgCl_2_, 10 mM HEPES, 1 mM EGTA, 140 mM D-mannitol, and 2 mM ATP). The external isotonic bath solution contained (in mM): 70 NaCl, 0.5 MgCl_2_, 2 CaCl_2_, 10 HEPES, and 140 D-mannitol. The 47% hypotonic bath solution was obtained by omitting D-mannitol from the solution, giving an osmolarity of 160 mosmol/L (47% hypotonicity, compared with the isotonic solution).

For the intracellular dialysis experiments, as described elsewhere [57], NPPB or tamoxifen was added to the pipette solution at a final concentration of 200 μM or 40 μM. A 20-min period was allowed for dialysis after breakthrough into the whole-cell configuration. After this dialysis period, the cells were unclamped and exposed to medium containing EGF (10 ng/ml) at 37^0^C for 5 min.

### Cell Migration Assay

*In vitro* wound-scratch experiments were performed to assess migratory potential as previously described [58]. In short, cells were seeded in 24-well plates and allowed to grow to confluence. Confluent monolayers were scratched with a 200-μl pipette tip. The cells were then cultured in the medium along with EGF (10 ng/ml) for 48 h. The images were recorded using a photomicroscope (Leica DFC950 camera; Leica Microsystems, Wetzlar, Germany) and cell migration was quantitated using Scion Image software (beta 4.0.2, Scion, Frederick, MD).

### Tracking of Endocytic Recycling β1 Integrin

Internalization and recycling of β1 integrin was observed according to previous protocols [35, 59]. To follow the endocytic pool of β1 integrin, cells were grown on glass coverslips and serum-starved overnight in DMEM containing 0.01% bovine serum albumin (BSA). 12G10 anti-β1 integrin (10 μg/ml, Abcam) antibody was added to cells at 4^0^C for 1 h for binding to surface integrin. Excess antibody was washed out using cold DMEM (with 0.01% BSA). Cells then were incubated at 37^0^C for 2 h to induce internalization. Surface antibodies were dissociated by an acid rinse (0.5% acetic acid, 0.5 M NaCl, pH 3.0) for 45 s. For recycling, cells were subsequently stimulated with prewarmed DMEM containing 20% FBS and 0.01% BSA at 37°C for 1 h, and then fixed with 4% (v/v) paraformaldehyde (PFA) in phosphate-buffered saline (PBS) for Immunofluorescence and confocal laser-scanning microscope observation. For flow cytometry analysis to quantify β1 integrin recycling, trypsinized and pelleted cells with recycling labeled integrin were incubated under non-permeabilizing conditions with Alexa Fluor 488-conjugated goat anti-mouse secondary antibody for 30 min, and fixed in 4% PFA/PBS. β1 integrin on the plasma membrane was determined by flow cytometry analysis (BD Biosciences, San Jose, CA).

### RNAi

To identify and to follow up the cells with knockdown of ClC-3 or CK18 expression, the vector pGPU6/GFP-ClC-3 shRNA (sh-ClC-3) that encodes shRNA and targets the specific sequence (ClC-3: 5′- GAGUAAAGUAGGAUGGCUUUCAACCCA-3′[60]; CK18: 5′- TATCACACGACTGCAGCTG-3′) was constructed and identiﬁed at GenePharma (GenePharma. Co., Ltd, Shanghai, China). The control vector sh-NC containing a non-silencing scrambled sequence was also constructed. Sh-RNA and sh-NC were transfected into cells by FuGENE HD (Roche, Indianapolis, IN). For high rate of transfection, the siRNA (small interfering RNA) for ClC-3 targeted to the same sequence and the NC (negative control) siRNA were synthesized and purified by GenePharma. SiRNA and si-NC were transfected into cells with the HiPerFect transfection reagent (Qiagen, Valencia, CA). For stable knockdown of ClC-3 in HeLa cells, the ClC-3 shRNA construct was cloned into a pLLU2G lentiviral vector, and the lentivirus was produced in Cyagen (Cyagen Bioscience Inc., Guangzhou, China).

### Co-Immunoprecipitation and Subcellular Fractionation

In order to eliminate the effects of antibody heavy and light chains, Co-immunoprecipation experiments were performed using the Pierce Co-Immunoprecipitation Kit (26149, Pierce; Rockford, IL) according to the manufacturer's instructions. Santa Cruz ClC-3 antibody (B-21: sc-133466) and Abcam ClC-3 antibody (ab86192) were used to immunoprecipitate and immunoblot respectively. 50 μg of the primary antibody was immobilized on the affinity columns and then protein lysates were pre-cleared with the control resin to minimize nonspecific binding. These lysates were loaded onto columns containing immobilized antibodies covalently linked to an amine-active resin and incubated under constant agitation for 12 h at 4°C. Equal volumes of the lysates were also applied to columns containing control resin and processed the same as the antibody coupling resin for negative controls. The co-immunoprecipitate was then eluted and analyzed by SDS-PAGE and Western blotting along with the input controls. Subcellular fractions were obtained with a Subcellular Protein Fractionation kit (Thermo Fisher Scientific, Waltham, MA) according to the manufacturer's protocol.

### Human Tissue Specimens, Tissue Microarrays and Immunohistochemistry

Tissues were collected following surgical resection at the Cancer Center of Guangzhou Medical University. Ethics approval for research using human tissue was obtained from the Guangzhou Medical University and included a waiver for consent. Tissue microarrays were purchased from US Biomax (MD, USA) and Outdo Biotech (Shanghai, China). Immunostaining was done on the tissue or tissue microarray using the MaxVision™ two-step systems (KIT-5010; Maixin Biotechnology Co., Ltd., Fuzhou, China) following the manufacturer's protocol. The intensity and distribution of the specific immunohistochemical staining reaction was evaluated using a semi-quantitative method (IRS-score), as previously described [61].

### Animal Experiments

All animal studies were conducted with institutional Animal Care and Use Committee approval. Transgenic mice overexpressing the human gene *CLCN3* were generated by oocyte microinjection at Cyagen Biosciences (Guangzhou, China). *CLCN3* gene was cloned into the pLV.Des3d.P/neo vector by Gateway recombinational cloning technology. The plasmid was then linearized and independently microinjected into fertilized mouse oocytes. Embryos that survived from microinjection were implanted into the oviduct of pseudopregnant female mice. Transgenic founders were identified via PCR of tail DNA. Overexpression of ClC-3 in the MMTV-PyMT spontaneous mammary tumor model was obtained by crossing ClC-3 females with MMTV-PyMT males. MMTV-PyMT and ClC-3/MMTV-PyMT female mice were monitored and sacrificed at different times for evaluating metastasis.

Approximately 1.0 × 10^7^ tumor cells in 100 μL serum-free medium were implanted in the subcutaneous tissue of the right abdominal wall of immunodeficient nude mice. Mice were killed when the diameter of tumor reached about 1.5 cm and axillary lymph nodes were observed and collected. For liver orthotopic implantation, MHCC97H cells were injected in the right flank of the mice. Once the subcutaneous tumor reached 0.7 cm in diameter, it was removed and cut into pieces about 2×2×2 mm, which were implanted into the liver of nude mice as previously described [62]. Five weeks later, the mice were sacrificed, and their lungs were fixed in Bouin's solution. Metastatic colonies were counted using a dissecting microscope. For injection in to the tail vein of mice, 3 × 10^5^ tumor cells suspended in 100 μl of PBS solution were injected. Three weeks after injection, the animals were sacrificed, and the lungs were then sampled and fixed for macro- and microscopic observation.

### Site-directed mutagenesis

To obtain amino acid exchange of K18 (Ser52) and K18 (Ser33), site-directed mutagenesis with K18–eGFP fusion vector was performed to replace Ser52 and Ser33 with alanine at GeneCopoeia (GeneCopoeia, Guangzhou, China). Tyrosine residues Y342 were mutated to phenylalanine by site-directed mutagenesis with ClC-3–eGFP fusion vector at GeneCopoeia. The entire coding sequence of each mutant was confirmed by sequencing.

### Statistical analysis

Differences between experiment groups were analyzed by Mann–Whitney U test or Student t test. The survival curves were plotted according to Kaplan-Meier method and checked by log-rank test. Data were presented as the mean ± SEM. All statistical tests were two-sided, and *P* <0.05 was considered statistically significant.

## SUPPLEMENTARY MATERIAL, FIGURES AND MOVIES






