# Supplementary material for: Chloride channel-3 promotes tumor metastasis by regulating membrane ruffling and is associated with poor survival
Source: Oncotarget. 2014 Dec 11;6(4):2434–50. doi: 10.18632/oncotarget.2966 (PMC4385862; doi:10.18632/oncotarget.2966)
Supplement: Supplementary file 1 [file oncotarget-06-2434-s001.pdf]

Chloride channel-3 promotes tumor metastasis by regulating membrane ruffling and is associated with poor survival

Supplementary Material

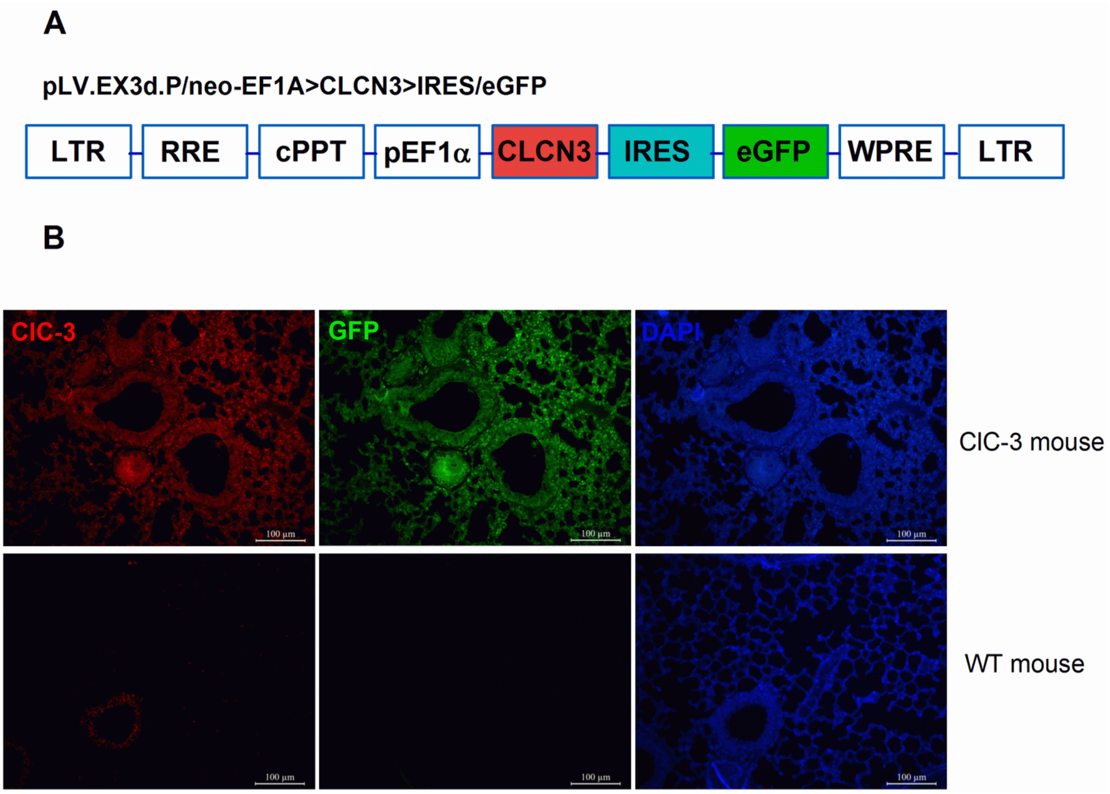

Figure S1: Generation of ClC-3 Transgenic Mice.

(A) Schematic diagram of the pLV.Des3d.P/neo-based constructs. IRES, internal ribosome entry site.

(B) Expression of the ClC-3 in lung tissues of wild-type and ClC-3 transgenic mice revealed by immunofluorescence.

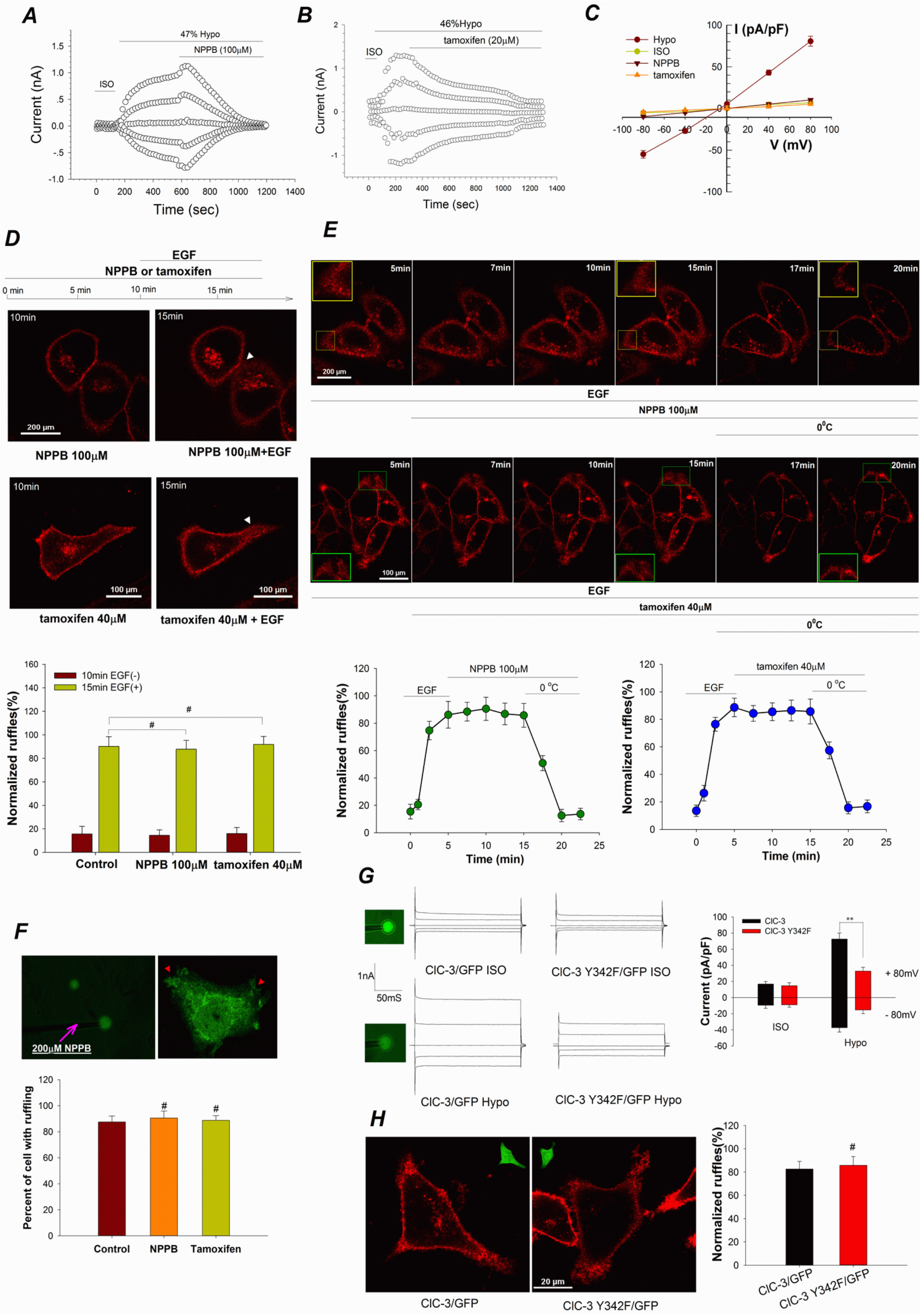

Figure S2: Regulation of Membrane Ruffle Formation by ClC-3 Is Independent of its Volume-activated Cl$^-$ Channel Properties

(A-E) Chloride channel blocker NPPB or tamoxifen inhibits volume-activated chloride currents but does not prevent (D) or abate (E) membrane ruffling induced by EGF. (A and B) The typical time

courses of activation of $I_{Cl,vol}$ and inhibition of the currents by extracellular NPPB (A,100μM) and tamoxifen (B, 40μM). (C) The current–voltage (*I*–*V*) relationship is presented (n = 5). ISO: isotonic solution; 47% Hypo: 160 mosmol/l hypotonic solution. Data shown are mean ± SEM. (D) Chloride channel blocker NPPB or tamoxifen does not prevent membrane ruffling （white arrows）induced by EGF. After 10 min treatment with NPPB or tamoxifen on Alexa Fluor-555 CTXB labeled cells, the cells was incubated in medium containing EGF (10 ng/ml) at 37^0C for 5 min. (E) Chloride channel blocker NPPB or tamoxifen does not abate membrane ruffling induced by EGF. After 5 min EGF stimulation the fluorescence labeled cells was incubated in medium containing NPPB or tamoxifen for 15 min and by following 5 min 0 ^{0}C treatments. # P >0.05; n=3 with >10 cells. Data are mean ± SEM. (F) Effects of blocking of cytoplasmic Cl^- current by intracellular dialysis of blocker NPPB on membrane ruffling (red arrows) of HeLa cells transfected with GFP vectors. NPPB or tamoxfein was added to the pipette solution at a final concentration of 200 μM or 40μM. After a 20-min dialysis period, the cells were unclamped and exposed to medium containing EGF at 37^0C for 5 min. # P >0.05 *VS* control; n=5.

(G and H) Effects of non-phosphorytable tyrosine mutation (Y342F) on ClC-3 chloride currents and membrane ruffling. (G) Representative whole-cell Cl^- currents and mean current densities measured at ±80 mV from HeLa cells transfected with GFP-tagged non-phosphorylatable mutant ClC-3 (Y342F). **P<0.01; n=4. (H) Representative photographs of HeLa cells transfected with Y342F mutant were stimulated with EGF. The cells were stained with Alexa Fluor-555 CTXB to observe ruffling (white arrows). # P >0.05 *VS* control; n=3 with >15 cells.

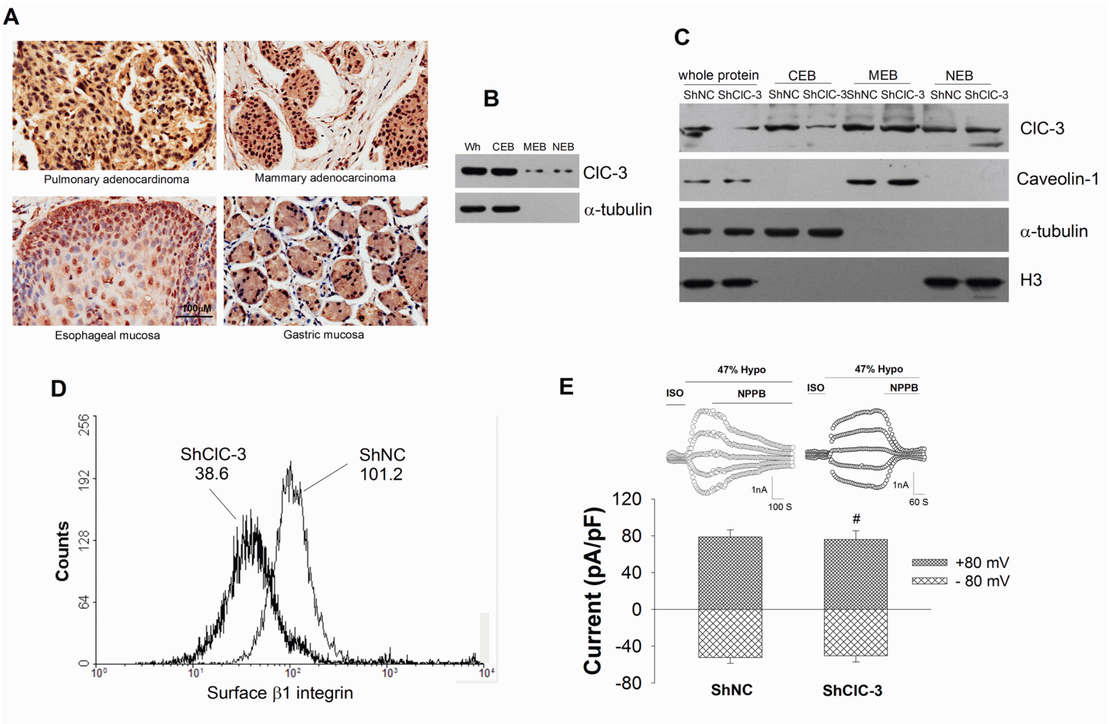

Figure S3: Effects of Down-regulation of Cytoplasmic ClC-3 Expression on Volume-activated Cl$^-$ Currents.

(A) Immunohistochemical staining of ClC-3 in lung and breast cancer and stomach and esophagus epithelium indicates that ClC-3 is also expressed in the nucleus.

(B) Western blot analysis and subcellular fractionation of HeLa cells. CE = cytoplasmic extract; ME = membrane extract; NE = nuclear extract.

(C) Partly stable down-regulation of ClC-3 expression by treating with lentiviral shRNA vectors targeted to ClC-3 does not change membrane ClC-3 expression.

(D) Down-regulation of cytoplasmic ClC-3 expression inhibits the recycling of β1 integrin to the plasma membrane. β1-Integrin recycling to plasma membrane in live HeLa cells with partly stable down-regulation of ClC-3 expression (shClC-3) quantitatively measured by a flow cytometry recycling assay. *P*<0.05 *vs* shNC, n=3.

(E) Down-regulation of cytoplasmic ClC-3 expression does not affect volume-activated Cl$^-$ currents. # *P* >0.05 *vs* shNC, n=4. Data are mean ± SEM.

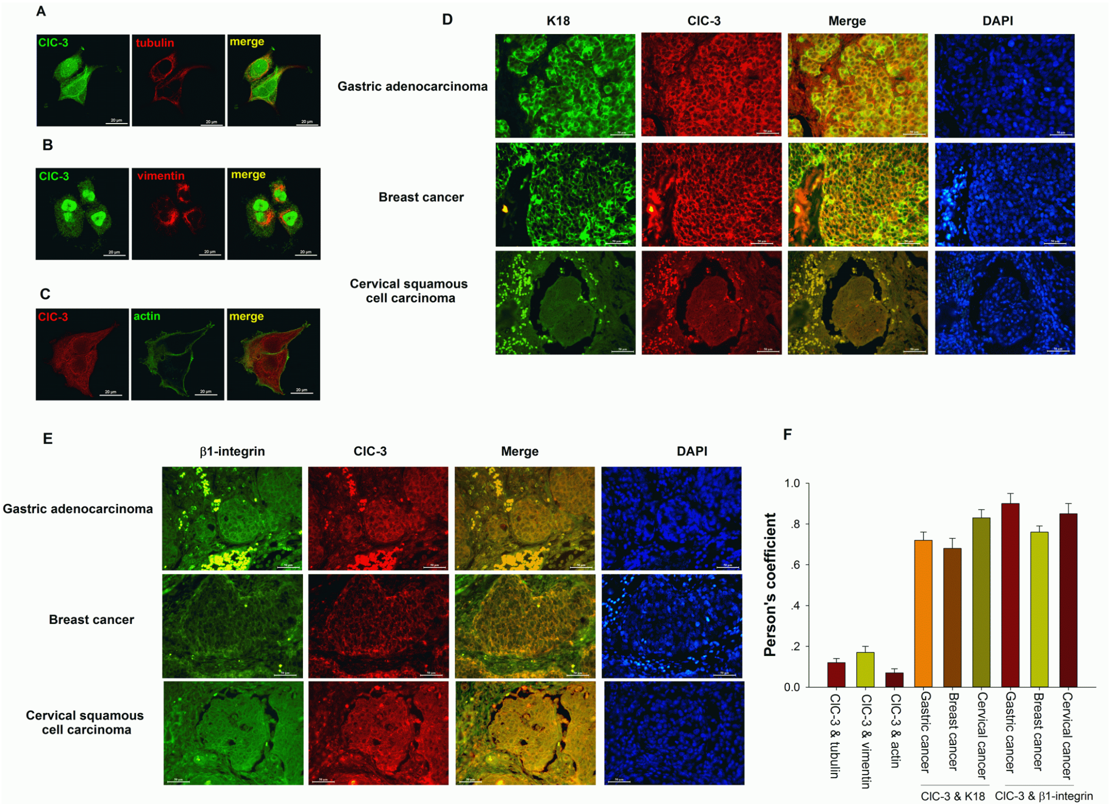

Figure S4: Colocalization Analysis of ClC-3 and Cytoskeleton in Ruffling Cells or β1 Integrin and CK18 in Different Types of Cancer Tissues

(A-C) ClC-3 does not colocalize with microtubules (A), vimentin (B) and microfilaments (C) in cytoplasm of HeLa cells with membrane ruffling. EGF-stimulated cells were fixed and incubated with different antibody for colocalization analysis.

(D and E) Immuno-colocalization analysis for the expression of ClC-3 and β1 integrin (D) or ClC-3 and CK18 (E) in human cancer tissues from stomach, breast, and cervix.

(F) A Pearson's coefficient was calculated to estimate the degree of co-localization of between the target proteins in cells or cancer tissues. The data was obtained from three independent experiments and presented as mean ± SEM.

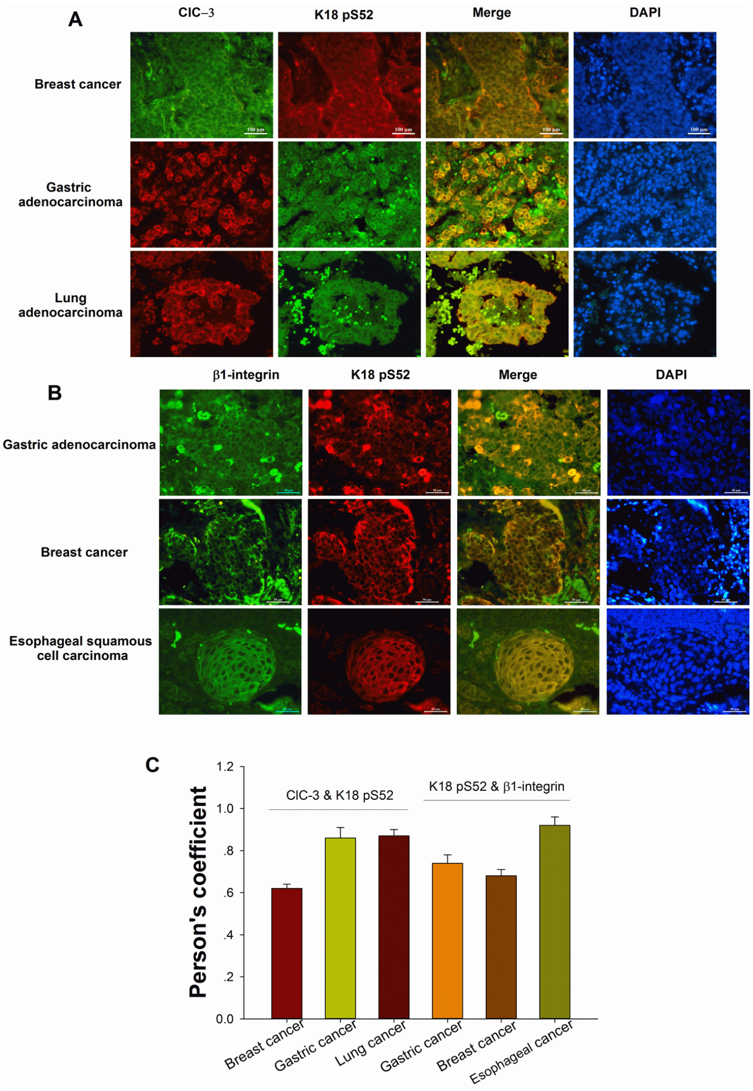

Figure S5. Analysis of ClC-3 or β1 Integrin Colocalization with K18 pS52 in Cancer Tissues

(A) Immuno-colocalization analysis for the expression of ClC-3 and K18 pS52 in cancer human tissues from stomach, breast, and Lung.

(B) Immunofluorescence staining for β1 integrin and K18 pS52 in stomach, breast, and esophagus caner tissues.

(C) A Pearson's coefficient was calculated to estimate the degree of co-localization of between ClC-3 and K18 pS52 orβ1 integrin and K18 pS52 in cancer tissues. The data was obtained from three independent experiments and presented as mean ± SEM.

Supplemental Movie Legends

Movie S1, Related to Figure 3. 3D Reconstruction of ClC-3 Subcellular Localization in HeLa Cells Stimulated by EGF.

ClC-3 was labeled with anti-ClC-3 and Alexa 488. The movie is a reconstruction of the data stack represented in Fig. 3B.

Movie S2, Related to Figure 8. 3D View of CK18 pS52 Subcellular Localization on HeLa Cells Stimulated by EGF.

CK18 pS52 was stained with anti-CK18 pS52 and Alexa 488. The movie is a reconstruction of the data stack represented in Fig. 8D.
